# Towards Identifying and Reducing the Bias of Disease Information Extracted from Search Engine Data

**DOI:** 10.1371/journal.pcbi.1004876

**Published:** 2016-06-06

**Authors:** Da-Cang Huang, Jin-Feng Wang, Ji-Xia Huang, Daniel Z. Sui, Hong-Yan Zhang, Mao-Gui Hu, Cheng-Dong Xu

**Affiliations:** 1 State Key Laboratory of Resources and Environmental Information System, Institute of Geographic Science and Natural Resource Research, Chinese Academy of Sciences, Beijing, China; 2 Key Laboratory of Surveillance and Early Warning on Infectious Disease, Chinese Center for Disease Control and Prevention, Beijing, China; 3 University of Chinese Academy of Sciences, Beijing, China; 4 College of Forestry, Beijing Forestry University, Beijing, China; 5 Department of Geography, The Ohio State University, Columbus, Ohio, United States of America; 6 School of Geographical Science, Northeast Normal University, Changchun, China; Ecole Polytechnique Federale de Lausanne, SWITZERLAND

## Abstract

The estimation of disease prevalence in online search engine data (e.g., Google Flu Trends (GFT)) has received a considerable amount of scholarly and public attention in recent years. While the utility of search engine data for disease surveillance has been demonstrated, the scientific community still seeks ways to identify and reduce biases that are embedded in search engine data. The primary goal of this study is to explore new ways of improving the accuracy of disease prevalence estimations by combining traditional disease data with search engine data. A novel method, Biased Sentinel Hospital-based Area Disease Estimation (B-SHADE), is introduced to reduce search engine data bias from a geographical perspective. To monitor search trends on Hand, Foot and Mouth Disease (HFMD) in Guangdong Province, China, we tested our approach by selecting 11 keywords from the Baidu index platform, a Chinese big data analyst similar to GFT. The correlation between the number of real cases and the composite index was 0.8. After decomposing the composite index at the city level, we found that only 10 cities presented a correlation of close to 0.8 or higher. These cities were found to be more stable with respect to search volume, and they were selected as sample cities in order to estimate the search volume of the entire province. After the estimation, the correlation improved from 0.8 to 0.864. After fitting the revised search volume with historical cases, the mean absolute error was 11.19% lower than it was when the original search volume and historical cases were combined. To our knowledge, this is the first study to reduce search engine data bias levels through the use of rigorous spatial sampling strategies.

## Author Summary

Search engine data have been increasingly used to track the outbreak of diseases in recent years. Although these resources are free and are updated frequently, certain biases and weaknesses are embedded in search engine data, making their predictions inaccurate and even misleading. These inaccurate estimations may be caused by various factors, such as external interferences, the changing behaviors of online users, or biases inherent in the search engine data. Within a given region or province, different cities may suffer from these biases to varying degrees. Spatial sampling can serve as a useful method in addressing this problem. Using search data for Guangdong Province in China, this article illustrates the utility of rigorous spatial sampling techniques for reducing search engine data bias levels.

## Introduction

Search engine data analysts (e.g., Google Flu Trends and other products of search engine query data) have made it convenient for us to track disease-related trends more effectively [1, 2]. However, their limitations have attracted increasing attention from the broader scientific community [3, 4]. The accuracy of disease tracking mechanisms that use search engine data is affected by Internet use trends, by external interferences from the media and from government policies, and by frequently updated algorithms created by search engine companies [1, 3, 4]. Such problems have manifested in Google Flu Trends, which missed the first wave of the influenza A/H1N1 pandemic in 2009 and which overestimated peak flu levels during the 2012/2013 season [2, 4]. As a typical application of big data, search engine data have attracted considerable public attention. Despite these biases and problems, as a new and alternative data source, we cannot deny the advantages of search engine data for monitoring diseases. To date, search engine data have been used to track the flu [1, 5], dengue fever [6, 7], Hand, Foot and Mouth Disease (HFMD) [8, 9] and several other diseases. Lazer et al. [3] have argued that search engine data, such as those used in Google Flu Trends and in other major data sets, should not be used alone but should instead be used as a supplement to traditional data. The integrated use of search engine data with conventional data sources has been proven to increase the accuracy of disease predictions [3, 6, 10].

In monitoring disease trends in a particular geographic area, previous studies have typically assessed the search volume (e.g., via Google Trends) of several keywords [5, 11–13]. Only a few studies have accounted for the internal spatial structure of an area, and most studies have not attempted to reduce search engine data biases levels. To complement previous studies, in this paper we use data related to HFMD in addressing three questions: (1) Is there a spatial difference in the search volume and search behaviors of HFMD? (2) Can historical cases of HFMD be used to reduce web data bias levels and to improve the relationship between search volume and real cases? (3) Can search engine data serve as a suitable tool for tracking HFMD trends in our pilot study area, Guangdong Province?

To answer these questions, the Baidu Index, a large data analyst index managed by Baidu (the largest Internet search engine company in China, making it the Chinese equivalent to Google), was used to collect search data on different scales that relate to HFMD in Guangdong Province. Based on the search behaviors of online users, keywords were divided into three groups to analyze the correlations they have with historical HFMD cases.

From historical cases on HFMD, a new Biased Sentinel Hospital-based Area Disease Estimation (B-SHADE) method was used to estimate the weight of each sample city, reducing biases in the search volume. In this case, we did not actually use sentinel hospital data but rather we applied the method using city-based search behavior data to function as “hospitals.” Thus, using the search volume of the sample city and the weight of each city that was estimated using B-SHADE, the area search volume was revised to achieve the best linear unbiased estimation. Finally, models were fitted by integrating historical HFMD cases and the revised search volume to examine the predictive effects of the revised search volume.

## Methods

### Study Area

Located in southern China with a subtropical climate and high population density, Guangdong had an HFMD occurrence rate that was more than four times higher than the national average from 2009 to 2011 [14]. In addition, living in one of the most developed areas in China, 60.4% of Guangdong’s population had Internet access in 2011, which is far higher than the Chinese average level (38.3%) [15]. These two conditions made Guangdong a suitable site for our study. The cities in Guangdong Province fall into two categories: cities located in the Pearl River Delta (PRD) region, which are more developed, and cities located outside of the PRD (OutPRD), which are less developed.

### HFMD Data

HFMD is a common human syndrome caused by highly contagious intestinal enteroviruses of the Picornaviridae family, which typically affects infants and children but also occasionally occurs in adults [16]. Numerous HFMD cases have been observed in the West Pacific region (including Japan, Malaysia, Vietnam, Singapore and China) [17–21]. From 2008–2012, 7,200,092 cases of HFMD were observed in China, and 2,457 (0.03%) of these cases were fatal [17]. As HFMD is highly contagious, its effective monitoring and control can significantly reduce its threat to public health in China. The government has spent a significant amount of money and time on HFMD monitoring.

In this study, HFMD cases were provided by the China CDC from January 1, 2009, to December 27, 2011. Reports of HFMD have been recorded through a national surveillance system for infectious diseases since May of 2008. Using a standard form, each district is required to report any cases of HFMD to this system daily [14, 17]. The reported information includes details about each patient (i.e., sex, birthday, home address, date of symptom onset, and date of diagnosis). Although reporting gaps or biases can result during the data collection process, this system is the country’s leading authority on HFMD data. As individuals are most likely to search for information on the disease immediately after one of their children has fallen ill, in our study, daily cases in each city were counted based on one attribute: the date of symptom onset. Weekly cases were then counted by adding up the number of daily cases every seven days. Throughout the study, historical HFMD cases were used as a reference standard to facilitate the selection of correlated search engine data. Only the number of cases is presented here, and we thus use and present no information on individual cases.

### Search Engine Data

The traditional monitoring system for HFMD and for other diseases relies upon a public health network that is expensive and often complex [22]. In addition, it typically takes approximately two weeks before the monitoring system’s formal report of its syndromic data is made public [23]. As a complement to the existing monitoring method, crowdsourced tracking and web data mining systems provide detailed and near-real-time information on diseases that is accessible at a marginal cost [4, 24]. Crowdsourced tracking systems call upon ordinary citizens to report on diseases that they encounter firsthand [25–30]. Alternatively, web data mining techniques are used to mine valuable disease information from the Internet, which has proven to be effective at monitoring diseases. For example, disease-related information is gathered by machines in order to better understand and record data in online systems [31, 32], disease-related keywords that individuals use in search engines are monitored [1, 5, 11, 33, 34], and text information posted on social media sites that relates to an individual getting sick is mined and analyzed [35–37]. Of all of the resources currently available, the use of search engine query data, which can be easily accessed through products such as Google Trends and the Baidu Index, is the most common method used to monitor diseases.

Our main research objects come from the Baidu index (http://index.baidu.com/), which is a platform that shares searching behaviors on Baidu search engine users. Baidu has a market share of more than 80% in China [11], making it better suited than Google to model the search behaviors of Chinese web users. The Baidu Index provides data on three spatial scales: country-, province- and city-wide. To date, the Baidu Index has been used to track influenza [6] and H7N9 [8] trends in China. However, previous studies have primarily focused on a single spatial scale [1, 11–13]. In this study, however, to better understand the relationship between HFMD cases and search volume levels, the search index of 11 keywords (Table 1) that are most highly correlated with HFMD are collected at the provincial and municipal scales. We collect our search terms using the collection process described by Yuan et al. [11]. We identify keywords by not only considering HFMD’s morbidity information but also by selecting recommended words from the following website: http://tool.chinaz.com/baidu/words.aspx. This website provides search engine optimization services and can identify the keywords most frequently used by Internet users in China.

**Table 1:** Keywords most highly correlated with HFMD cases.

General Keywords	Corr	Treatment Keywords	Corr	Prevention Keywords	Corr
HFMD symptoms	0.803	Ev71 virus	0.719	How to prevent HFMD	0.706
What medicines should HFMD (patients) take	0.757	Ev71	0.602	The guide to preventing and treating HFMD	0.69
What medicines should HFMD (patients) use	0.645	Enterovirus	0.601	The guide to preventing and controlling HFMD	0.62
Papule	0.678				
HFMD	0.603				

Note: The keyword names are based on English-language keyword meanings. The Chinese names can be found in S2 Table. “Corr” denotes the maximum cross Pearson correlation coefficient (0–7 weeks before and after the current period) between the HFMD cases and the search keyword index.

We select our keywords using the following three steps. The first step involves filtering the keywords by combining the website’s recommended keywords with the morbidity of HFMD, causing non-disease-related keywords to be excluded. In the second step, keywords with a Pearson correlation coefficient of more than 0.4 are selected. In the last step, to ensure the keywords do not have lag effects in monitoring HFMD, we examine cross correlations between the keywords and the HFMD cases that last 0–7 weeks, both before and after the current period. Finally, 11 keywords are found to have a maximum cross correlation with the HFMD cases of more than 0.6. These data are then processed into weekly data and, based on their attributes, are classified into three groups: General Keywords, Prevention Keywords and Treatment Keywords (see S1 Text for more detail). The composite index is counted by adding these three types of keywords together. S2 Fig presents actual HFMD cases and the Baidu Index for the study period. The largest number of HFMD cases and the largest composite index are found in the Pearl River Delta.

### B-SHADE

B-SHADE (Biased Sentinel Hospital-based Area Disease Estimation) is a method that was originally designed to generate space-time population disease estimates from biased hospital records [38]. In using a weighted summation of biased sentinel hospital records, area diseases can be counted to achieve an unbiased and minimal level of error variance.

We intend to estimate the entire province’s unbiased search volume by using cities with less bias in their search volume records. Due to the existence of cities with biased search volume levels, historical space-time information on search volumes between cities can no longer be used. In order to determine the historical space-time structures of HFMD trends between cites, we assume that the prevalence of HFMD may also be related to HFMD online search trends. This assumption is based on the high correlation between the selected keywords and HFMD cases. In our study, the composite index of most cites has a correlation of more than 0.7, making it possible to determine the spatial-temporal structure of search volume based on historical cases.

**Fig 1:**
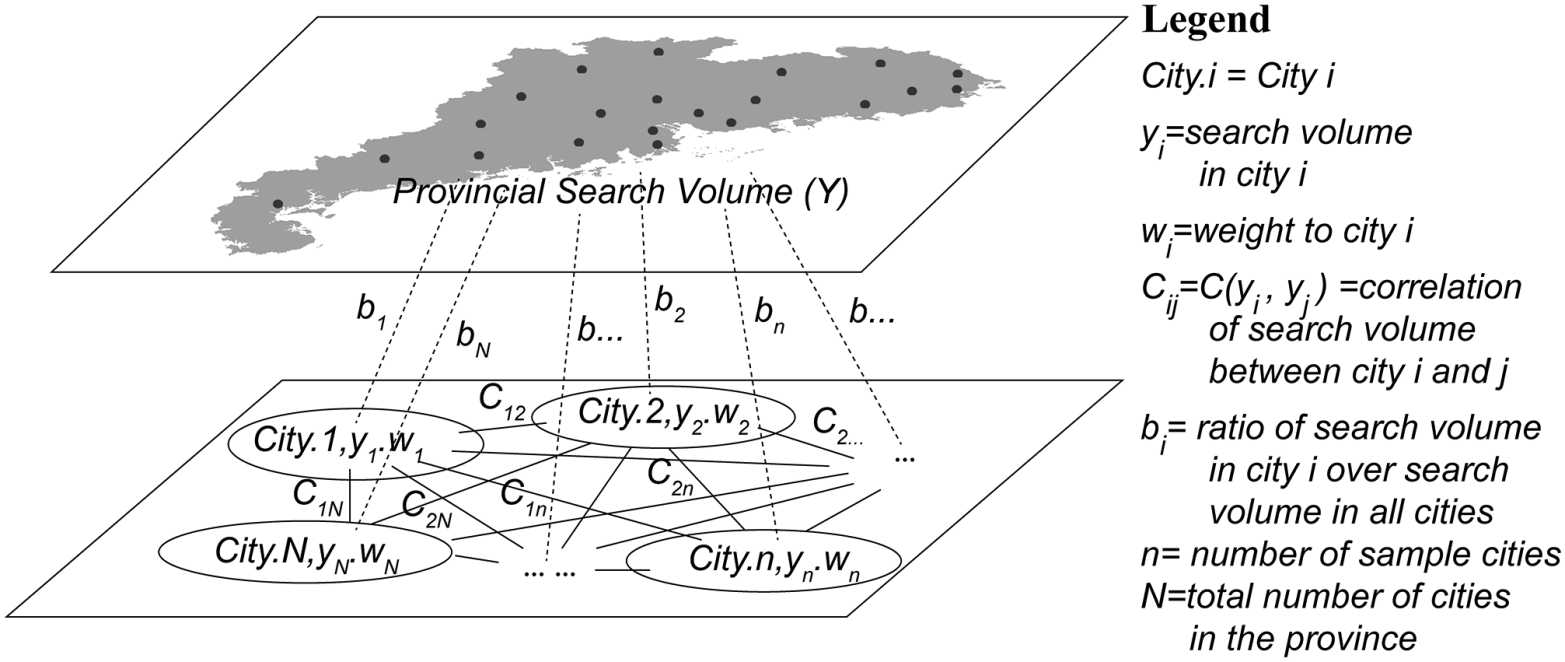
The relationship between cities and provincial search volumes [38]. The solid lines represent the horizontal search trend relationships (covariances) between the cities, which can be determined from historical HFMD cases; the dashed lines represent vertical search trend links (ratios) between each city and the entire province. Spatial sampling of all cities is conducted to select cities with high quality search engine data. Finally, the search volume of all of the cities can be estimated based on the sampled cites and quantified relationships. The map on the top was created in ArcGIS 10.2 (Environmental Systems Resource Institute, ArcScene Release 10.2, ESRI, Redlands, California).

B-SHADE can be calculated as follows. First, historical cases can be used to obtain the horizontal relationship (covariance) between cities and vertical links (ratio) between each city and the entire province [38] (see Fig 1). Next, the weights of each sampled city are calibrated to minimize the error variance of the estimated area search volume under the constraints of the best linear unbiased estimation (BLUE). As a result, the potential bias for the entire province is removed, and the new estimated provincial search volume is better suited for subsequent use.

The weight of each sample city is calculated to reach the BLUE [38, 39]:

$$\begin{array}{l} \sum_{j=1}^{n}w_{j}C\left(y_{i},y_{j}\right)+\mu b_{i}=\sum_{j=1}^{N}C\left(y_{i},y_{j}\right)\\ \sum_{i=1}^{n}w_{i}b_{i}=1,b_{i}=E\left(y_{i}\right)/E\left(Y\right) \end{array}$$ (1)

where *n* is the number of sample cities, *N* represents the total number of cities, *w* denotes the weight of each sample, *C*(*y*_*i*_,*y*_*j*_) is the covariance between the *ith* and *jth* cities obtained from the HFMD cases, *b*_*i*_ represents the rate of cases between the sample city and the province, and *μ* represents a Lagrange multiplier.

From the calculation results of *w* and *μ*, the estimated population and error variance are determined using the following equation [38, 39]:

$$\begin{array}{l} y_{w}=\sum_{i=1}^{n}w_{i}y_{i}\\ \sigma_{y_{w}}^{2}=\left(r_{n}-1\right)\sum_{i=1}^{n}\sum_{j=1}^{n}w_{i}w_{j}C\left(y_{i},y_{j}\right)-2\mu \end{array}$$ (2)

Where

$$r_{n}=\sum_{i=1}^{N}\sum_{j=1}^{N}C\left(y_{i},y_{j}\right)/\sum_{i=1}^{n}\sum_{j=1}^{n}w_{i}w_{j}C(y_{i},y_{j}).$$

B-SHADE was developed as a free software: B-SHADE Estimation and Sampling (http://www.sssampling.org/B-shade/) [39], which is designed as a graphical interface for ease of calculation.

### Predictive Models

Log-linear models are fitted with Eqs (3) and (4) to examine the predictive capacities of the original composite index and historical cases of HFMD that were obtained from the China CDC. Our goal is to predict HFMD trends in real time by combining historical HFMD cases with the search index, both before and after making revisions. Autoregressive models are thus fitted with (Eq 5) to compare the predictive results of the original composite index and the revised index.

$$Log(\text{rea}{\text{l}}_{t})=\alpha_{0}+\alpha_{1}Log(\text{inde}{\text{x}}_{t})+\varepsilon_{t}$$ (3)

$$Log(\text{rea}{\text{l}}_{t})=\beta_{0}+\beta_{1}Log(\text{rea}{\text{l}}_{t-2})+\beta_{2}Log(\text{rea}{\text{l}}_{t-3})+\beta_{3}Log(\text{rea}{\text{l}}_{t-4})+\phi_{t}$$ (4)

$$Log(\text{rea}{\text{l}}_{t})=\chi_{0}+\chi_{1}Log(\text{rea}{\text{l}}_{t-2})+\chi_{2}Log(\text{inde}{\text{x}}_{t})+\lambda_{t}$$ (5)

where real_*t*_, real_*t*−2_, real_*t*−3_ and real_*t*−4_ are cases of HFMD for the current week and for two, three and four weeks prior, respectively; index_*t*_ denotes the search index (original composite index or revised index) of the present week; *α*_0_, *α*_1_, *β*_0_, *β*_1_, *β*_2_, *β*_0_, *χ*_0_, *χ*_1_, and *χ*_2_ are the coefficients; and *ε*_*t*_, *ϕ*_*t*_, and *λ*_*t*_ are the residuals.

## Results

### Regional Analysis of Different Keywords

**Fig 2:**
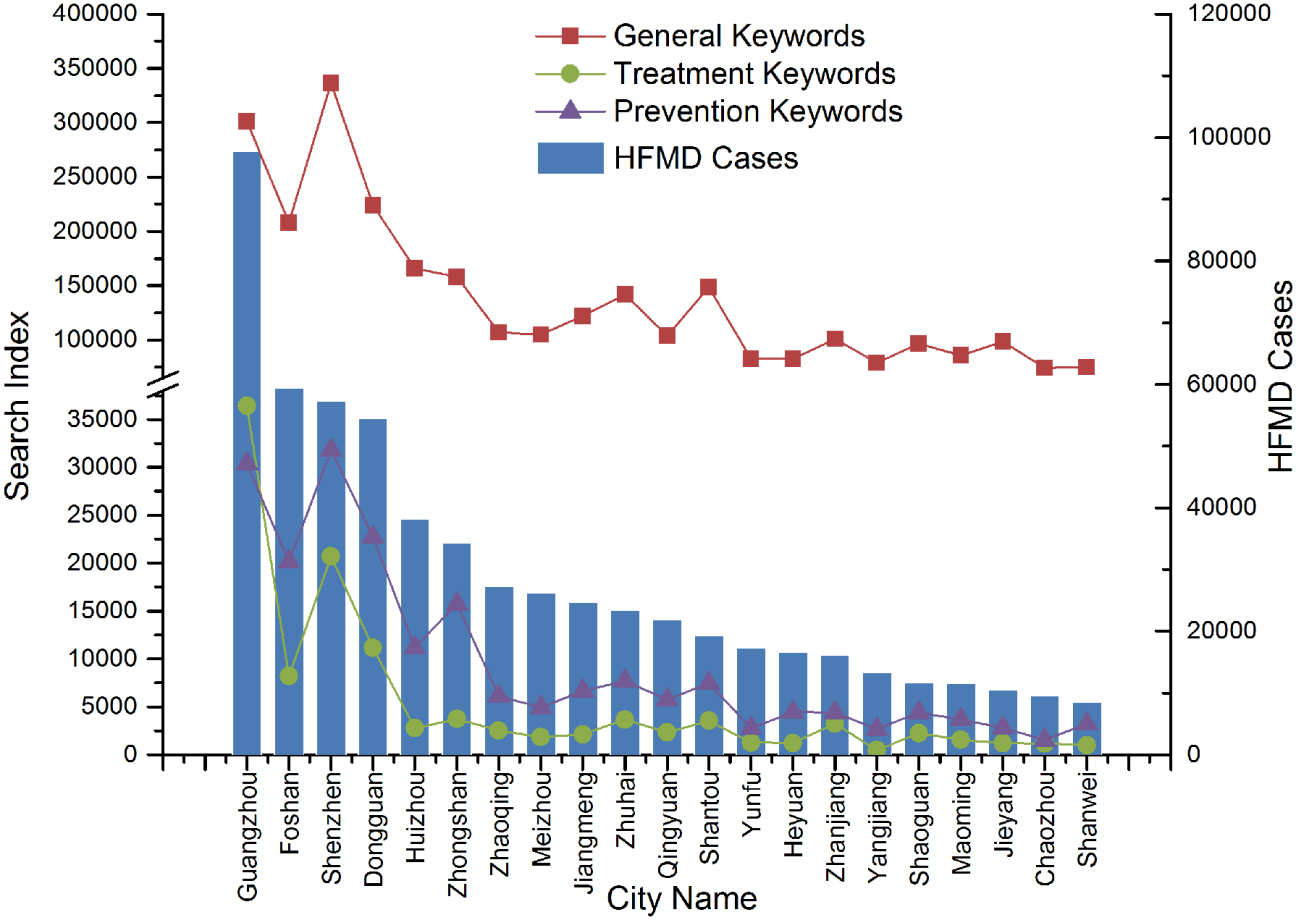
Comparisons of real cases and the three types of keywords across the province from 2009 to 2011. The histogram shows the total number of HFMD cases for every city; the line charts represent the search volumes of general keywords (red), treatment keywords (green), and prevention keywords (purple) for all of the cities.

In order to analyze the spatial distributions of different types of keywords across the province, the total search volume for each type of keyword was first determined at the city level. The results show that the search volumes of each type of keyword have a similar spatial distribution across the entire province. Cities suffering from more HFMD cases had a higher search volume for all types of keywords (Fig 2 and S2 Fig), while cities showing fewer HFMD cases had a lower search volume for all types of keywords. General keywords had a much higher search volume than keywords related to treatment and prevention. This result was expected, as individuals typically use words that are easy to understand and that are widely used.

**Fig 3:**
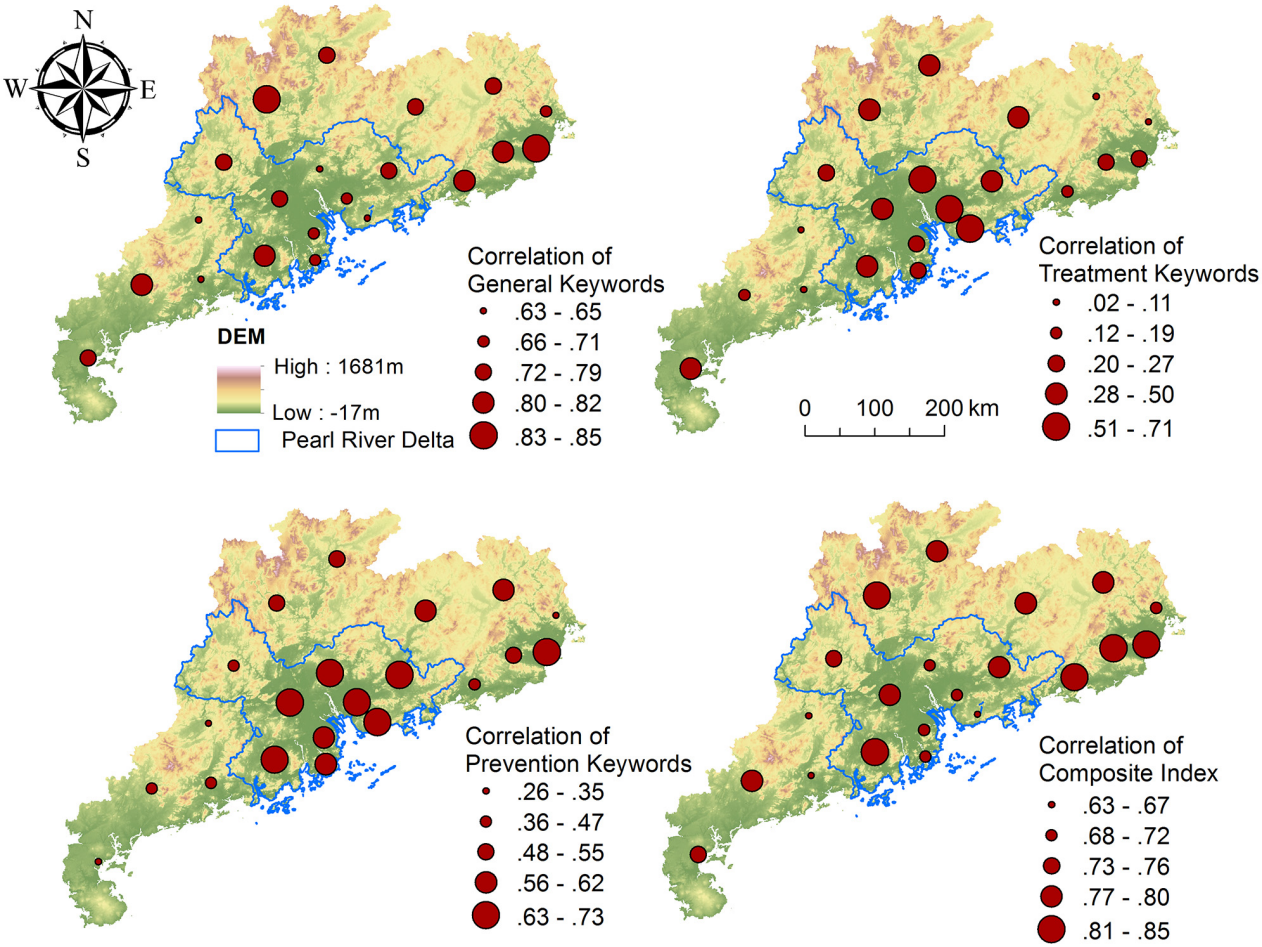
Correlations between HFMD cases and search volumes across the province from 2009 to 2011. The map on the top left shows the spatial distribution of the Pearson correlation coefficient for the HFMD cases and general keywords from 2009 to 2011 for all of the cities. The same indicators were measured for treatment keywords (top right), prevention keywords (bottom left), and composite indexes (bottom right). Maps were created in ArcGIS 10.2 (Environmental Systems Resource Institute, ArcMap Release 10.2, ESRI, Redlands, California).

However, when we compared the correlations between each group of keywords and real cases at the city and provincial levels, the strength of the correlations was found to vary throughout the province. We hypothesized that a higher search volume in a city would lead to higher correlations among all groups of keywords. However, we found that developed cities that are located within the PRD showed a stronger correlation with treatment and prevention keywords, whereas compared to cities located outside of the delta region, they had a relatively weak correlation with general keywords and the composite index (Fig 3). By contrast, cities located outside of the PRD showed a weaker correlation with treatment and prevention keywords, whereas they showed a relatively stronger correlation with general keywords and the composite index. This phenomenon implies that search behaviors related to HFMD vary throughout the province.

### Sample City Selection and the Revised Results of B-SHADE

As described above, treatment and prevention keywords were better suited to describe trends in the PRD region, whereas general keywords and the composite index best simulated HFMD trends in the OutPRD region. However, the number of general keywords and the composite index value were much larger than those of treatment and prevention keywords. Therefore, we cannot ignore their influence on the simulation of HFMD trends. The composite index reflected the total search volume of a city, and although biases are present, we use the B-SHADE method to reduce bias levels and to render the dataset more suitable for use.

The total correlation between real cases and the composite index of the 21 cities is shown in S3 Table. The correlation for the entire province was 0.8. B-SHADE was used to reduce the search volume bias for the entire province. By using an appropriate number of sampled cities to recount the search volume for the entire province, the newly counted search volume (defined as the revised index) could better simulate HFMD trends for the entire province.

**Fig 4:**
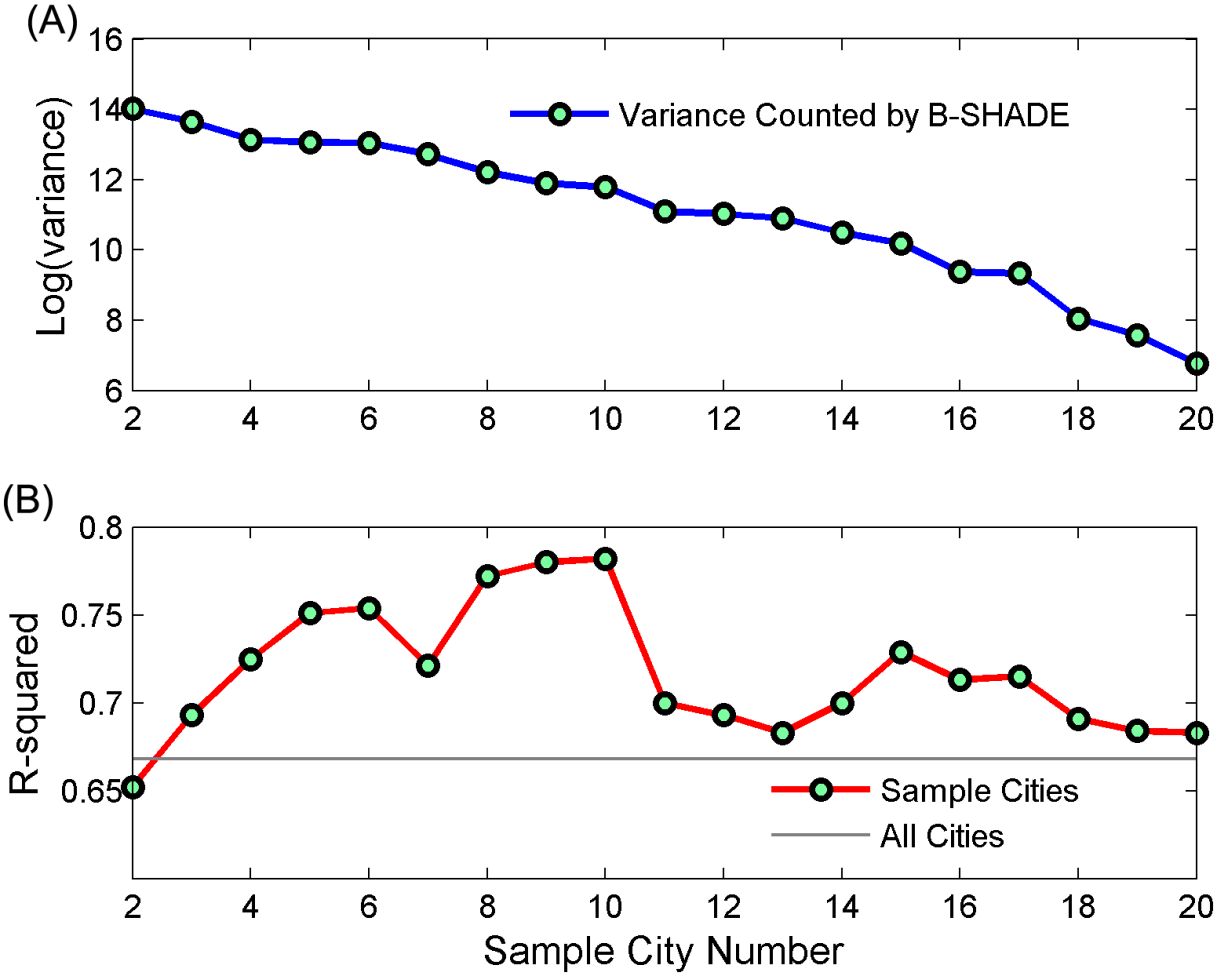
Estimating results from a different number of sample cities. (A) A comparison of the variance determined by B-SHADE using different sample cities; (B) The performance of the revised search index for different sample cities. The red line denotes the fitting results of different sample cities, and the baseline (gray line) denotes the fitting result for all of the cities (without sampling).

The combination of sample cities was selected from 21 cities. To select the best combination, between 2 and 20 sample cities were added. We added cities based on the ordering of their correlations (from high to low), allowing us to compare their variances as determined through B-SHADE in addition to the performance of the revised index’s fitting results. We fit the model by using (Eq 3) to observe when the revised index best explained real cases. Fig 4 shows that the variance determined by B-SHADE decreased as the number of sample cities increased; however, the best (Eq 3) fitting result was obtained when 10 cities were used. Compared to the original search volume, which used 21 cities without sampling, the revised index represents the output of the combination of cities with the high-quality search volume and valuable information obtained from historical cases. Thus, the revised index can predict HFMD cases more reliably.

The 10 cities selected present correlations that were higher than those of the other cities. For the real cases, the revised index showed a correlation of 0.864, which was also larger than the original correlation (0.8).

### Prediction Results

To examine the predictive capacities of the original composite index, the revised index, the official historical cases and the historical cases combined with original and revised indices, we used all of the previous data to predict cases of HFMD for a given week. To validate the accuracy of the predictions, they were compared to the official HFMD cases. From the start of 2009 to the end of 2011, this method was used on a rolling basis to predict HFMD cases week by week, thus reflecting how such a system would likely be used in the real world [3]. As there are not sufficient data available for historical data determinations for the first ten weeks of 2009 (less than ten weeks), their HFMD cases are not predicted.

Models were fitted to Eqs (3), (4) and (5).

The original composite index was fitted using (Eq 3):

$$Log(\text{rea}{\text{l}}_{t})=\alpha_{0}+\alpha_{1}Log(\text{CompositeInde}{\text{x}}_{t})+\varepsilon_{t}$$

The revised index was fitted using (Eq 3):

$$Log(\text{rea}{\text{l}}_{t})=\alpha_{0}+\alpha_{1}Log(\text{RevisedInde}{\text{x}}_{t})+\varepsilon_{t}$$

The historical cases (lagged CCDC) were fitted using (Eq 4):

$$Log(\text{rea}{\text{l}}_{t})=\beta_{0}+\beta_{1}Log(\text{rea}{\text{l}}_{t-2})+\beta_{2}Log(\text{rea}{\text{l}}_{t-3})+\beta_{3}Log(\text{rea}{\text{l}}_{t-4})+\phi_{t}$$

The combination of historical cases and the composite index (composite index + lagged CCDC) were fitted using (Eq 5):

$$Log(\text{rea}{\text{l}}_{t})=\chi_{0}+\chi_{1}Log(\text{rea}{\text{l}}_{t-2})+\chi_{2}Log(\text{CompositeInde}{\text{x}}_{t})+\lambda_{t}$$

The combination of historical cases and the revised index (revised index + lagged CCDC) were fitted using (Eq 5):

$$Log(\text{rea}{\text{l}}_{t})=\chi_{0}+\chi_{1}Log(\text{rea}{\text{l}}_{t-2})+\chi_{2}Log(\text{RevisedInde}{\text{x}}_{t})+\lambda_{t}$$

The performances of these models were measured using the mean absolute error (MAE), mean absolute percentage error (MAPE), and root-mean-squared error (RMSE). Table 2 summarizes the accuracy metrics for each of these models. Of all of the models examined, the model that combined the revised index and lagged CCDC performed the best. Compared to the composite index models, revised index, lagged CCDC, and combined composite index and lagged CCDC, we found MAE improvements of 47.34%, 35.35%, 21.53% and 11.19%, respectively. An MAE of 749.2 denotes that each city’s error level reaches nearly 35.68 cases per week. Improving total HFMD case estimations may help the government and hospitals better prepare for an impending epidemic.

**Table 2:** Comparisons of different models for the estimation of HFMD cases.

Model	MAE	MAPE	RMSE
Composite Index	1422.7	40.11	1872.2
Revised Index	1158.9	31.13	1595.1
Lagged CCDC	954.8	26.93	1537
Composite Index + Lagged CCDC	843.6	23.5	1200.3
Revised Index+ Lagged CCDC	749.2	20.81	1067.6

Note: The MAE, MAPE and RMSE were counted from the 11^th^ week of 2009 to the end of 2011. CCDC stands for the Chinese Center for Disease Control and Prevention.

**Fig 5:**
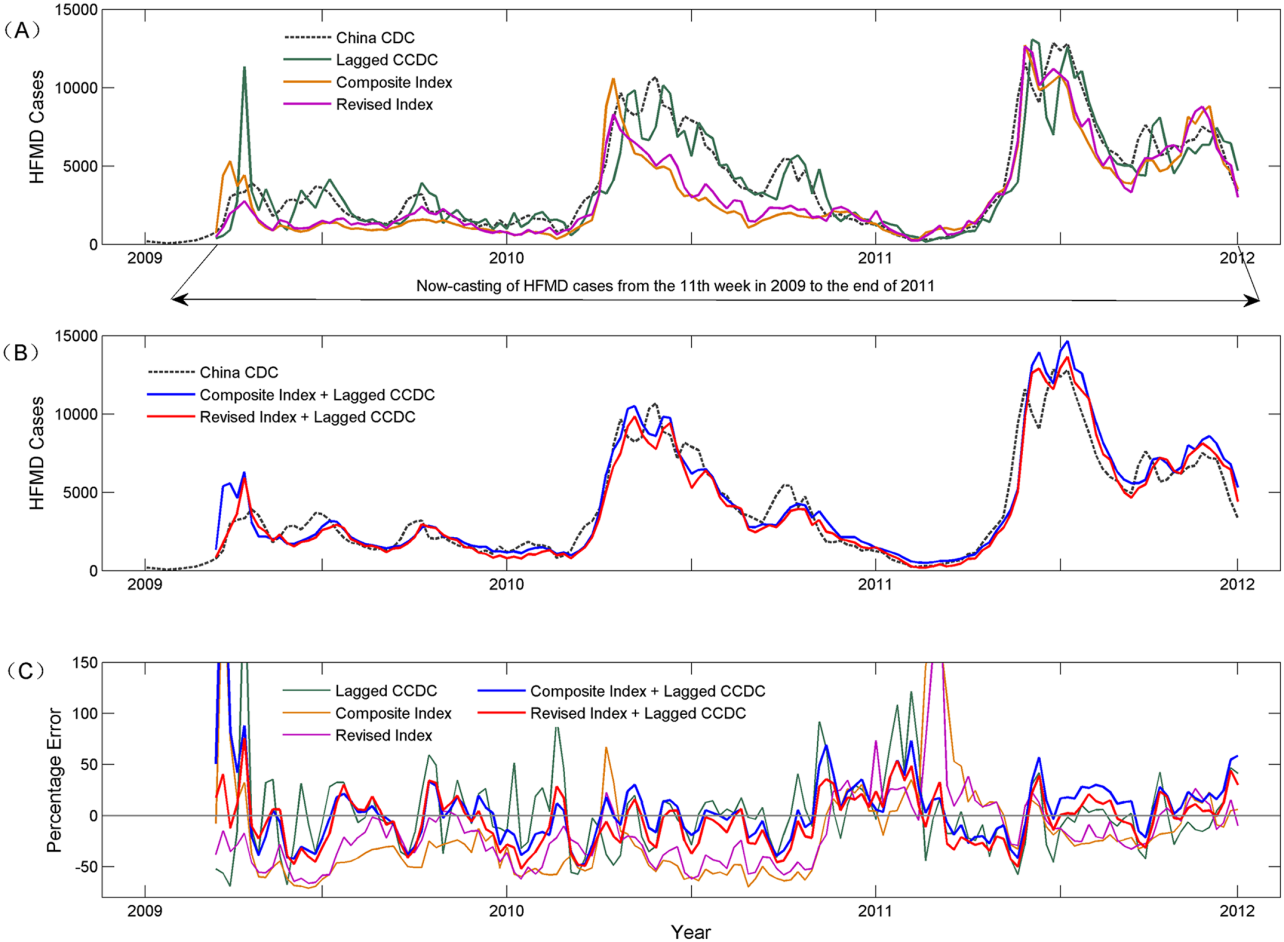
The predicting curves of HFMD trends and their percent error levels. The dashed line (China CDC) represents real cases of HFMD. (A) Comparison between the prediction results based strictly on historical cases (Lagged CCDC; green line), the composite index (orange line), and the revised index (magenta line); (B) Comparison between the prediction results of the combined composite index and historical cases (composite index + lagged CCDC; blue line) and the combined revised index and historical cases (revised index + lagged CCDC; red line); (C) Comparison between the percent errors of these five models.

Fig 5 provides a visualization of the predicting curves of all of the models, illustrating that the combined revised index and lagged CCDC is more stable and accurate in modeling HFMD trends. The mean absolute percent error was 20.81%, which was lower than both the lagged CCDC (26.93%) and the combined composite index and lagged CCDC (23.5%).

## Discussion

In recent years, search engine-based systems of disease surveillance have developed rapidly [22]. Although such systems have proven to be efficient and convenient to use compared to traditional monitoring systems, they still present numerous problems and limitations. Several complex factors have prevented search engine data from serving as reliable surveillance tools [2–4]. Google has stopped publishing current estimates of GFT online, although researchers who wish to use the latest GFT data can still ask Google for access. Previous studies have reported on various weaknesses of search engine data; however, few of these studies have attempted to address these issues or to effectively improve prediction accuracy levels. In our study, we introduce the B-SHADE method as a means of using historical cases to improve the usability and stability of search engine data. In consideration of not only relationships between cities but also of the ratio of each city’s size throughout a province, in estimating the search volume of an entire province, ten cities with the highest correlations with real cases were selected as sample cities. Both the correlations and predictive accuracy levels found were improvements from those of the original data. Thus, this research increased the usefulness of the Baidu Index as a tool for monitoring the spread of HFMD in Guangdong Province.

Unlike influenza, which is easily transmitted to individuals of all ages and which is associated with a variety of symptoms, the primary sufferers of HFMD are children under the age of 5. Thus, individuals who search online for HFMD information are primarily parents and teachers, and the keywords that they use are often relatively specific. Ginsberg et al. [5] used 45 significant keywords that were automatically selected from 50 million words to identify influenza epidemics in the U.S.; the 45 keywords were found to focus on 13 topics. In our study, 11 keywords were selected to monitor search trends related to HFMD, and we classified these keywords into 3 groups based on their meanings. We found that cities in the Pearl River Delta, which show the highest number of HFMD cases, also generate the highest search volume of HFMD keywords. Conversely, cities outside of the Pearl River Delta region show fewer HFMD cases and a lower search volume. We found that web queries show a strong relationship with data obtained from traditional surveillance systems [1, 2, 5, 22, 40], echoing the findings of other studies.

We found the search behaviors of HFMD to vary spatially throughout Guangdong Province. Developed cities located within the PRD show a stronger correlation with treatment and prevention keywords whereas, compared to cities located outside of the delta region, their correlation with general keywords and the composite index is relatively low. This phenomenon may be due to superior medical devices and higher levels of education that characterize the PRD region in addition to the fact that developed cities include more hospital staff, researchers and teachers, all of whom are likely to use treatment and prevention keywords preferentially. The lower correlation between general keywords and the composite index for the developed cities may be attributable to the fact that general keywords, which are primarily used by ordinary people and by the media, can easily be disturbed by external factors and government policies. Cook et al. [41] suggested that search engine data should perform poorly for diseases that are subject to high media exposure (e.g., the H1N1 pandemic of 2009). HFMD, which is also a seasonal epidemic, always attracts media attention during its outbreak season, and it therefore presents biases in search engine data. Salathé et al. [40] also found that public interest in media reports will wane over time even if new cases continue to emerge. All of these uncertainties contribute to search engine data biases, particularly in terms of general keywords, which also heavily influence the accuracy of the composite index.

We found that historical cases of HFMD can be used to reduce search engine data biases and to improve their correlations with real cases. Harford [42] notes that during an opinion poll, it is more important to find an unbiased sample than to cover a large population. By properly selecting samples of the voting population, even 3,000 interviews can accurately predict a final voting result. As this case is similar to the voting problem, we sought to select cities with an unbiased search volume to predict the total search volume for the entire province. The original correlation for the entire province was found to be 0.8, but 10 cities showed a correlation of close to or larger than 0.8. When these cities were selected as sample cities to estimate the total search volume for the entire province (21 cities) via B-SHADE, the correlation improved from 0.8 to 0.864. Thus, the revised search volume was found to be more similar to the real cases.

By examining the predictive capacities of the newly revised search volume, we found that the revised search volume improves the predictive results of HFMD trends. This is attributable to the fact that the revised search volume takes advantage of historical relationships between HFMD cases for each city and of the highly precise search volumes of the sampled cities. After our revisions, the MAE was recorded as 749.2, signifying that each city’s error level represented approximately 35.68 cases per week. This result is not only better than the error level generated when strictly using historical HFMD cases, the composite index, and the revised index but rather it is also 11.19% lower than the error level generated when combining the composite index with lagged CCDC data. While Cayce et al. [8] found Google Trends to serve as a suitable tool for tracking HFMD trends in developed countries in Asia (e.g., Singapore and Japan), they also found that Google Trends is not strongly correlated with surveillance data in Hong Kong. This phenomenon may be more pronounced for developing countries where regional differences are significant. The spatial diversity of the regional economy and of Internet access levels has limited the capacities of search engine data in accurately tracking disease trends in developing countries and regions. By tracking a number of important cities from which high quality search engine data can be easily obtained, our method has the potential to help countries predict the total number of diseases cases (e.g., HFMD and influenza) to occur in advance (especially for countries that present significant regional differences).

However, this study still presents limitations. One limitation pertains to the fact that while the keywords that we used were determined based on their correlations with real cases, the search index of the keywords can be easily changed by the search behaviors of Internet users. Thus, to maintain high correlations with real cases, we must generate a dynamic word bank that can adapt to online research behavior variations. Another limitation relates to the fact that compared to traditional surveillance methods, search engine-based surveillance requires Internet access. According to the China Internet Network Information Center (CNNIC), the Internet was available to 48.8% of all Chinese citizens in June of 2015 [43]. While we assume that the guardians of children who suffer from HFMD search online for certain information, not every guardian is in the habit of conducting online information searches. Finally, our estimations of total search volume were based on the assumption that the historical space-time structure of HFMD prevalence can also describe online research trends on HFMD. This assumption is also dependent on guardian tendencies to search for online information when their children are ill.

Despite these limitations, we are the first to present a means of reducing Internet bias and of improving the stability of search engine data from a geographical perspective. With the rapid development of Internet services, we have entered an era of Big Data, and more traditional statistical approaches can be used to mine valuable information from large but biased datasets. The observation of such phenomena and the discovery of regularities within Big Data in making full use of these resources constitute new challenges to address.

## Supporting Information

S1 Text Information on keyword selection, the composition process and the GFT experiment. This document illustrates how we selected and composed the keywords. An experiment was conducted on GFT to observe the spatial distribution of correlations between GFT and the number of influenza-like illness cases occurring in the U.S. from 2003 to 2015.
(PDF)

S1 Fig Behaviors of the guardians of children who either suffer from or who are at risk of contracting HFMD. When individuals use the Internet to find related information, data are collected by Internet companies and are stored in an online database; when individuals go to hospitals, these cases are also recorded by doctors, resulting in the formation of historical cases. This figure was created in Microsoft Office Visio 2007.
(TIF)

S2 Fig Distribution of HFMD cases and the composite index for Guangdong Province (2009 to 2011). The small map on the top left shows the positioning of Guangdong Province in China. The large map shows the spatial distribution of HFMD cases (red dots) and the composite index (base map) for Guangdong Province. Maps were created in ArcGIS 10.2 (Environmental Systems Resource Institute, ArcMap Release 10.2, ESRI, Redlands, California).
(TIF)

S1 Table Keywords related to HFMD with Pearson correlation coefficients exceeding a value of 0.4. This table presents the Chinese and English names of keywords with a Pearson correlation coefficient of more than 0.4 for HFMD cases.
(PDF)

S2 Table Keywords related to HFMD with a maximum cross-correlation coefficient of more than 0.6. “Corr” denotes the Pearson correlation coefficient, “Max Cross Corr” is the maximum cross correlation between the HFMD cases and keyword search index, “Ahead Weeks” denotes the number of weeks ahead of the current week when the maximum cross correlation occurred (i.e., a negative number represents the number of weeks lagged behind the current week).
(PDF)

S3 Table The correlation of real cases and the composite index for 21 cities. This table shows the correlation between the composite index and HFMD cases for 21 cities from 2009 to 2011. The correlations of all of the cities are significant at the 0.01 level. Cities listed in bold are the selected sample cities.
(PDF)
