# Supplementary material for: Towards Identifying and Reducing the Bias of Disease Information Extracted from Search Engine Data
Source: PLoS Comput Biol. 2016 Jun 6;12(6):e1004876. doi: 10.1371/journal.pcbi.1004876 (PMC4894584; doi:10.1371/journal.pcbi.1004876)
Supplement: S1 Text — This document illustrates how we selected and composed the keywords. An experiment was conducted on GFT to observe the spatial distribution of correlations between GFT and the number of influenza-like illness cases occurring in the U.S. from 2003 to 2015. (PDF) [file pcbi.1004876.s001.pdf]

Supplemental Methods

1.1 Keyword Selection

Keywords were selected according to HFMD's basic morbidity information [1] and the words recommended by the website http://tool.chinaz.com/baidu/words.aspx. This website provides service for the search engine optimization and could tell us the most frequently used keywords searched by the web users in China. All the keywords were firstly selected on the provincial scale. We selected the keywords by following three steps. The first step is to filter the keywords by combining the keywords recommended by the website and the morbidity of HFMD. In this way, the non-disease-related keywords were excluded; in the second step, all the keywords were selected only if they have a Pearson correlation coefficient larger than 0.4 (S1 Table); in the third step, to ensure the keywords would not have lag effects in monitoring the HFMD, we filtered the keywords by setting an appropriate threshold [2]. By examining the cross correlation 0-7 weeks before and after the current period, 11 keywords were found to have a maximum cross correlation of more than 0.6 with the HFMD cases (S2 Table). The reason that we used a threshold of 0.6 was that some keywords with a correlation under 0.6 did not have enough search volume on the city scale. In addition, if we set a threshold of 0.65, the number of keywords was extremely limited (only 6), and some significant keywords would have been excluded, thereby hindering our forecasting accuracy. The selected 11 keywords in S2 Table represent the HFMD trend in Guangdong Province as a whole. The search index of these 11 keywords in the 21 cities of Guangdong Province was also compiled in this way.

1.2 Keyword Classification and Composition

When HFMD occurs, people turn to the Internet for help in finding related information. Guardians' behavior can be simulated, as shown in S1 Fig.. Most people use keywords based on what they remember from doctors, media reports and surrounding advertisements, which may or may not be accurate. At this stage, the keywords selected are general and broad, but they concentrate on several basic symptoms or potential treatments; these words are defined as General Keywords. When staff at hospitals and research institutes refer to the Internet for the causes and methods of treating HFMD using professional keywords, we define these types of keywords as Treatment Keywords. Finally, with respect to preventing HFMD, parents and schools tend to search online for information on how to prevent HFMD; these types of keywords are classified as Prevention Keywords.

The 11 keywords were then classified into 3 groups according to the analysis above (S2 Table). All keywords are counted according to the formula $index = \sum_{i=1}^{n} X_i^l$, where

the index represents the type of keywords to be counted, n represents the number of keywords contained in this group and l is the number of weeks ahead of current week when the maximum cross correlation occurred. The composite index was then counted by adding these three types of keywords together, that is:

$$composite\ index = general\ keywords + treatment\ keywords + prevention\ keywords \quad (1)$$

1.3 The spatial distribution of correlation between GFT and the amount of influenza like illness in US

GFT is available at https://www.google.org/flutrends/about/, the data were collected from 2003/9/28 to 2015/4/12. Data of influenza like illness were downloaded from the website of Centers for Disease Control and Prevention (http://www.cdc.gov/flu/weekly/fluactivitysurv.htm) within the same time period of GFT.

We then counted the correlation of GFT and the amount of influenza like illness in ten Health and Human Services（HHS）Regions in US from 2003 to 2015. The correlation ranged from 0.62 to 0.83 (S3 Fig.), which demonstrate that spatial bias does existed. However, it was puzzle that the whole nation's correlation is 0.846, which is higher than any of the regions. So it was not suitable to reduce the bias of GFT of the whole nation by simply using the data based on the scale of HHS regions. Cities with high correlations should be discovered in the further research to act as sample cities to solve this problem. It is also necessary for researchers to deeply analyze the hiding bias of GFT by mining the related keywords that form them both in space and time.

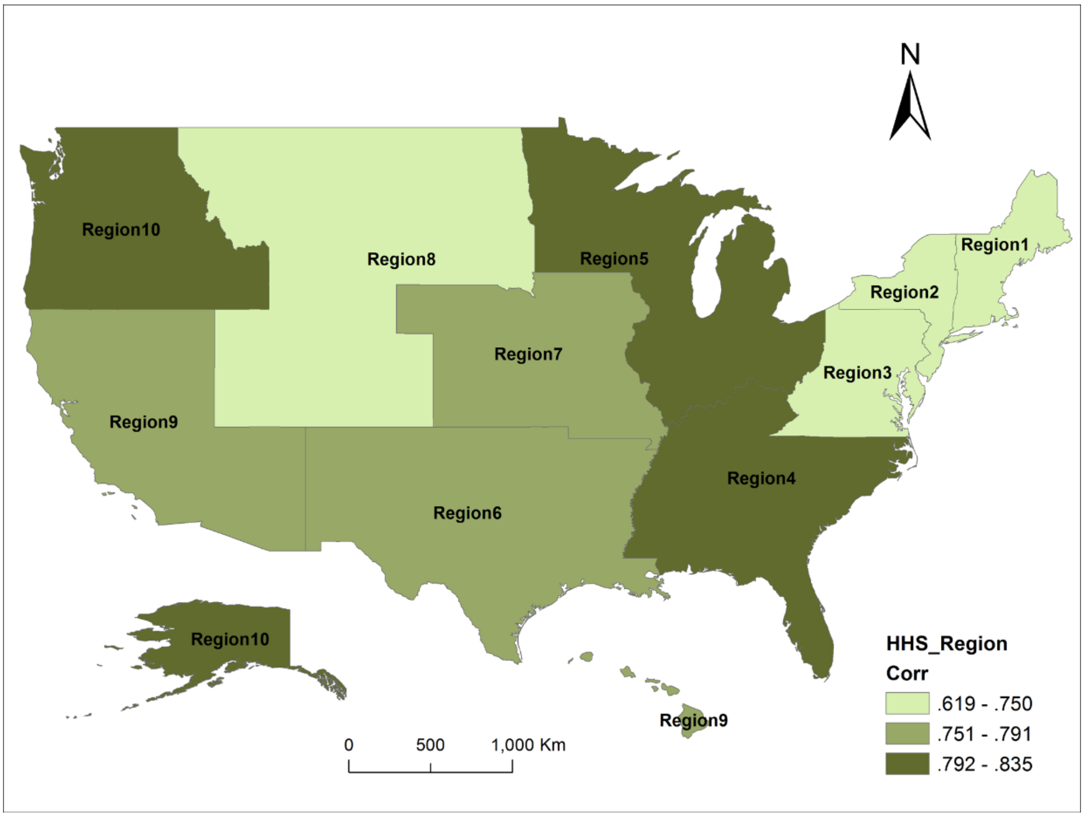

S3 Fig. The spatial distribution of correlation between GFT and the amount of influenza like illness in ten Health and Human Services（HHS）Regions in US from 2003 to 2015. This map was created in ArcGIS 10.2 (Environmental Systems Resource Institute, ArcMap Release 10.2, ESRI, Redlands, California).

References

1. World Health Organization (2011) A guide to clinical management and public health response for Hand, Foot and Mouth disease (HFMD). Available: http://iris.wpro.who.int/bitstream/handle/10665.1/5521/9789290615255_eng.pdf. Accessed 29 Oct 2014.
2. Yang X, Pan B, Evans JA, Lv B. Forecasting Chinese tourist volume with search engine data. Tourism Management. 2015; 46: 386-397.
