# Supplementary figures and images for: Towards Identifying and Reducing the Bias of Disease Information Extracted from Search Engine Data
Source: PLoS Comput Biol. 2016 Jun 6;12(6):e1004876. doi: 10.1371/journal.pcbi.1004876 (PMC4894584; doi:10.1371/journal.pcbi.1004876)

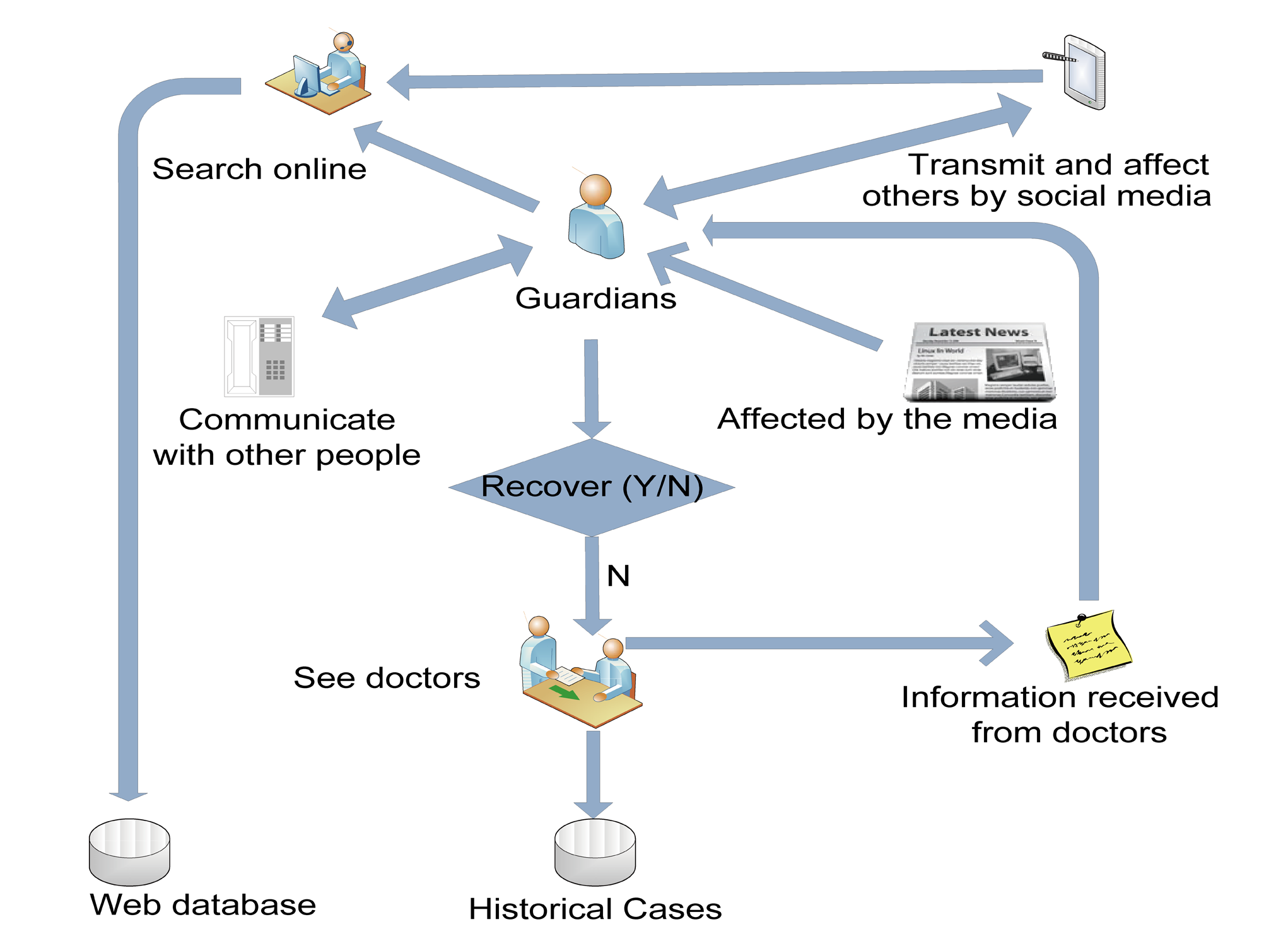

Supplement: S1 Fig — When individuals use the Internet to find related information, data are collected by Internet companies and are stored in an online database; when individuals go to hospitals, these cases are also recorded by doctors, resulting in the formation of historical cases. This figure was created in Microsoft Office Visio 2007. (TIF) [file pcbi.1004876.s002.tif]

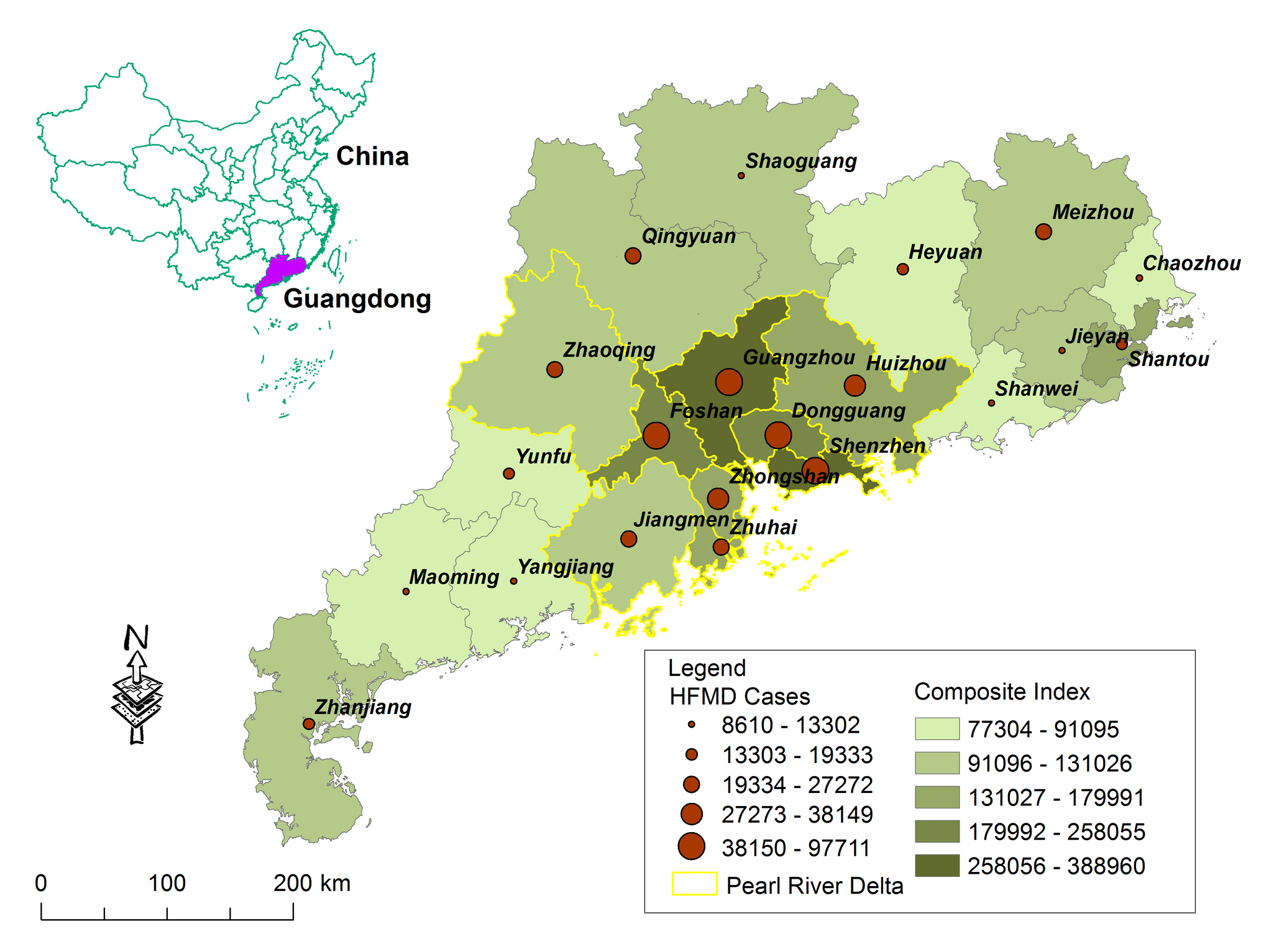

Supplement: S2 Fig — The small map on the top left shows the positioning of Guangdong Province in China. The large map shows the spatial distribution of HFMD cases (red dots) and the composite index (base map) for Guangdong Province. Maps were created in ArcGIS 10.2 (Environmental Systems Resource Institute, ArcMap Release 10.2, ESRI, Redlands, California). (TIF) [file pcbi.1004876.s003.tif]
