# Supplementary material for: Towards Identifying and Reducing the Bias of Disease Information Extracted from Search Engine Data
Source: PLoS Comput Biol. 2016 Jun 6;12(6):e1004876. doi: 10.1371/journal.pcbi.1004876 (PMC4894584; doi:10.1371/journal.pcbi.1004876)
Supplement: S1 Table — This table presents the Chinese and English names of keywords with a Pearson correlation coefficient of more than 0.4 for HFMD cases. (PDF) [file pcbi.1004876.s004.pdf]

Chinese	English	Chinese	English
手足口病症状	HFMD symptoms	手足口病	HFMD
手足口病吃什么药	What medicines should HFMD (patients) take	手足口病用什么药	What medicines should HFMD (patients) use
Ev71病毒	Ev71 virus	Ev71	Ev71
肠道病毒	Enterovirus	如何预防手足口病	How to prevent HFMD
手足口病防治指南	The guide to preventing and treating HFMD	手足口病预防控制指南	The guide to preventing and controlling HFMD
口腔溃疡	Mouth ulcer	疱疹	Herpes
儿童手足口病	HFMD in children	小儿手足口病	Pediatric HFMD
怎样预防手足口病	How to prevent HFMD	宝宝口腔溃疡怎么办	What should I do if (my) baby has a mouth ulcer
手足口病症状图片	Photos of HFMD symptoms	手足口病的预防	Prevention of HFMD
手足口病图片	Photos of HFMD	手足口病 症状	HFMD and its symptoms
手足口病特征	Characteristics of HFMD	皮疹	Rash
