# Supplementary material for: Towards Identifying and Reducing the Bias of Disease Information Extracted from Search Engine Data
Source: PLoS Comput Biol. 2016 Jun 6;12(6):e1004876. doi: 10.1371/journal.pcbi.1004876 (PMC4894584; doi:10.1371/journal.pcbi.1004876)
Supplement: S2 Table — “Corr” denotes the Pearson correlation coefficient, “Max Cross Corr” is the maximum cross correlation between the HFMD cases and keyword search index, “Ahead Weeks” denotes the number of weeks ahead of the current week when the maximum cross correlation occurred (i.e., a negative number represents the number of weeks lagged behind the current week). (PDF) [file pcbi.1004876.s005.pdf]

Keywords Class	Chinese	English Meaning	Original Corr	Max Cross Corr	Ahead Weeks
General Keywords	手足口病症状	HFMD symptoms	0.79	0.803	1
	手足口病吃什么药	What medicines should HFMD (patients) take	0.753	0.757	-1
	手足口病用什么药	What medicines should HFMD (patients) use	0.645	0.645	0
	丘疹	Papule	0.678	0.678	0
	手足口病	HFMD	0.587	0.603	1
Treatment Keywords	Ev71病毒	Ev71 virus	0.719	0.719	0
	Ev71	Ev71	0.572	0.602	1
	肠道病毒	Enterovirus	0.577	0.601	1
Prevention Keywords	如何预防手足口病	How to prevent HFMD	0.697	0.706	1
	手足口病防治指南	The guide to preventing and treating HFMD	0.678	0.69	1
	手足口病预防控制指南	The guide to preventing and controlling HFMD	0.456	0.62	5
