# Supplementary material for: Towards Identifying and Reducing the Bias of Disease Information Extracted from Search Engine Data
Source: PLoS Comput Biol. 2016 Jun 6;12(6):e1004876. doi: 10.1371/journal.pcbi.1004876 (PMC4894584; doi:10.1371/journal.pcbi.1004876)
Supplement: S3 Table — This table shows the correlation between the composite index and HFMD cases for 21 cities from 2009 to 2011. The correlations of all of the cities are significant at the 0.01 level. Cities listed in bold are the selected sample cities. (PDF) [file pcbi.1004876.s006.pdf]

| City | Corr | City | Corr | City | Corr |
|---|---|---|---|---|---|
| Guangzhou | 0.69 | Yunfu | 0.65 | **Shantou** | 0.85 |
| Foshan | 0.78 | Zhuhai | 0.72 | **Huizhou** | 0.79 |
| Yangjiang | 0.63 | Zhaoqing | 0.76 | **Heyuan** | 0.79 |
| Chaozhou | 0.70 | Zhongshan | 0.71 | **Jiangmeng** | 0.82 |
| Shenzheng | 0.67 | **Shaoguang** | 0.80 | **Meizhou** | 0.80 |
| Zhanjiang | 0.76 | **Maoming** | 0.79 | **Qingyuan** | 0.84 |
| Dongguang | 0.71 | **Shanwei** | 0.83 | **Jieyang** | 0.82 |
